# From bench to triage: diagnostic utility of delta-like canonical notch ligand-1 (DLL-1) for early sepsis prediction in the emergency department

**DOI:** 10.1007/s15010-026-02794-y

**Published:** 2026-04-13

**Authors:** Aurelia Hübner, Katharina Friedrich, Noa Galtung, Vivienne Theobald, Judith Schenz, Britta Hecke, Melanie Kraß, Markus A. Weigand, Kai Kappert, Wolfgang Bauer

**Affiliations:** 1 Department of Emergency Medicine, Charité − Universitätsmedizin Berlin, Corporate member of Freie Universität Berlin and Humboldt-Universität zu Berlin, Hindenburgdamm 30, 12203 Berlin, Germany; 2 Institute of Diagnostic Laboratory Medicine, Clinical Chemistry and Pathobiochemistry, Charité − Universitätsmedizin Berlin, Corporate member of Freie Universität Berlin and Humboldt-Universität zu Berlin, Augustenburger Platz 1, 13353 Berlin, Germany; 3 Department of Anesthesiology, Medical Faculty Heidelberg, Heidelberg University, Im Neuenheimer Feld 672, 69120 Heidelberg, Germany

**Keywords:** DLL-1, Sepsis, Infection, Emergency department

## Abstract

**Background:**

The early identification of patients with sepsis among those presenting with suspected infection in the emergency department (ED) remains challenging. Delta-like canonical Notch ligand-1 (DLL-1), a Notch pathway ligand involved in immune and endothelial signaling, has been shown to reflect disease severity and outcomes in ICU-based sepsis cohorts. Its value for early risk stratification at ED presentation is less well defined.

**Methods:**

We analyzed DLL-1 concentrations in serum samples from a prospectively enrolled ED cohort of adults presenting with clinically suspected acute infection. Blood samples were obtained during the initial clinical assessment. Sepsis was defined according to Sepsis-3 criteria and adjudicated by an expert panel, including evaluation of organ dysfunction within 72 h after presentation. DLL-1 concentrations were measured using enzyme-linked immunosorbent assay (ELISA), and diagnostic performance was assessed using AUROC analysis.

**Results:**

Among 300 enrolled patients, 74 (24.7%) were classified as having sepsis. DLL-1 concentrations were significantly higher in patients with sepsis than in those without sepsis (median 11,454 vs. 8,085 pg/mL; p < 0.001). DLL-1 demonstrated moderate discriminatory performance for sepsis at ED presentation (AUROC 0.69, 95% CI 0.62–0.76). Performance was lower than that of procalcitonin and NEWS2 but comparable to C-reactive protein and lactate. DLL-1 showed a moderate correlation with SOFA score. Importantly, correlations with CRP and white blood cell count were nonsignificant.

**Conclusions:**

When measured at ED presentation, DLL-1 concentrations are elevated in patients with sepsis but showed only moderate diagnostic performance. These findings are consistent with earlier disease stages and lower degrees of organ dysfunction compared with ICU populations. DLL-1 may offer complementary host-response information beyond established biomarkers, but does not support stand-alone use for early sepsis diagnosis and warrants further evaluation within multimodal risk stratification strategies.

Trial registration.

DRKS00017395.

**Supplementary Information:**

The online version contains supplementary material available at 10.1007/s15010-026-02794-y.

## Introduction

Delta-like canonical Notch ligand-1 (DLL-1) is a Notch pathway ligand that plays a pivotal role in intercellular communication, particularly in regulating immune responses. It has attracted increasing scientific interest due to its dual capacity to enhance protective immune activity against pathogens while also being linked to various pathological conditions, such as cancer and infectious diseases [1, 2]. In the context of bacterial infection, DLL-1 is released from activated monocytes and contributes to endothelial activation and increased vascular permeability, indicating its involvement in core pathophysiological processes of sepsis [3, 4]. These mechanisms strengthen the rationale for DLL-1 as a biomarker candidate for sepsis.

The majority of sepsis cases originate in the community and are therefore first encountered in the emergency department (ED), where early risk stratification is critical for patient outcomes [5–7]. However, standard clinical evaluation and sepsis screening tools primarily detect present organ dysfunction and may therefore identify sepsis only when decisive therapeutic interventions may already be delayed [8]. Patients presenting to the ED constitute a highly heterogeneous population with respect to causative pathogen, site and compartment of infection, age, comorbidities, and disease severity, ranging from early infection to septic shock. Consequently, ongoing efforts focus on biomarkers to address this diagnostic gap.

Although ICU-based studies suggest that DLL-1 reflects disease severity and predicts adverse outcomes in advanced sepsis [9], its role in early risk stratification among emergency department patients presenting with clinical suspicion of infection has not yet been assessed in great detail. Thus, this study investigated DLL-1 in a heterogeneous ED cohort with suspected infection. The objective was to evaluate the performance of DLL-1 in identifying sepsis or sepsis-related clinical deterioration when measured at the earliest possible time point during emergency care.

## Methods

### Study design and patient selection

**Fig. 1:**
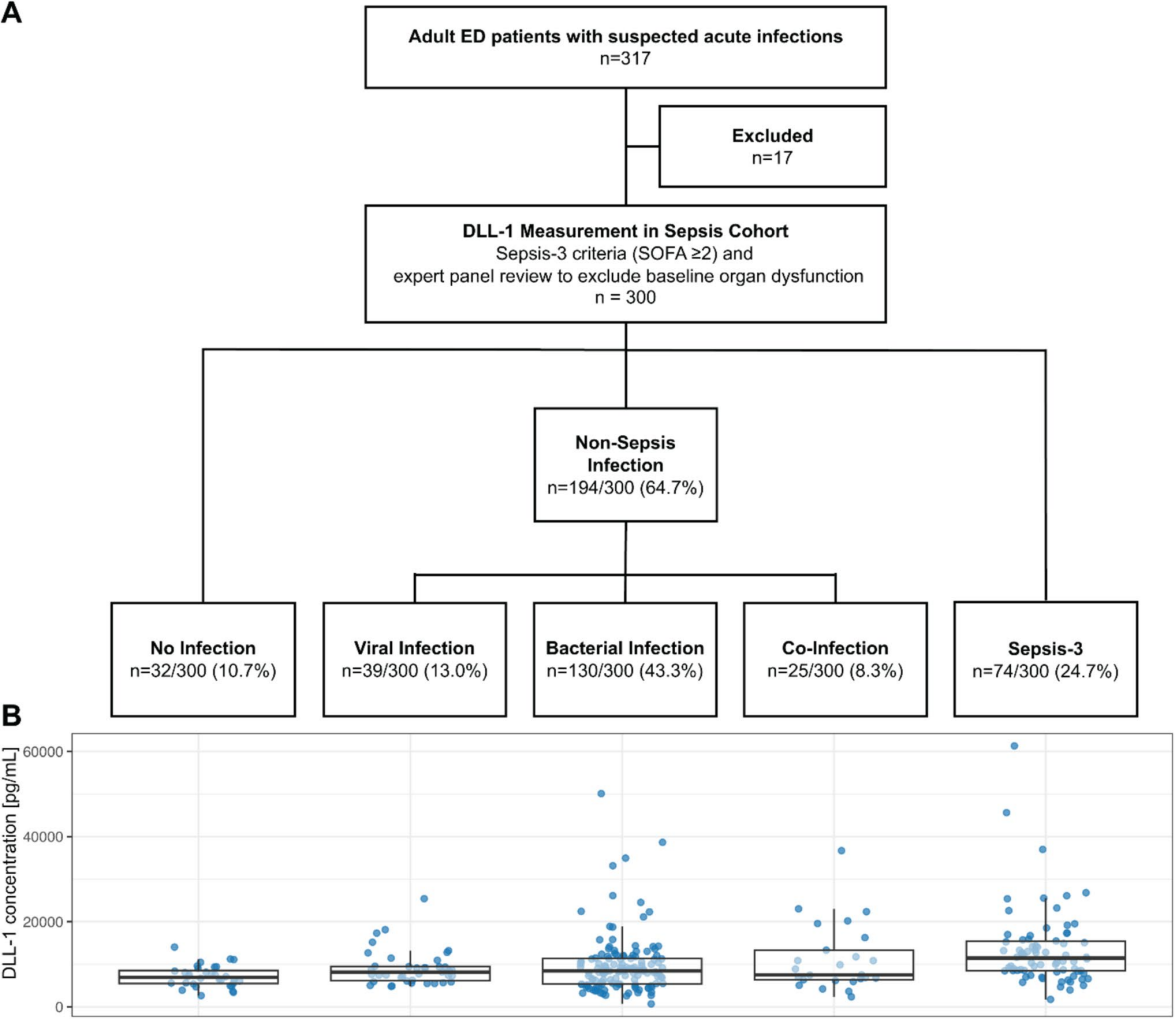
Study Design, Clinical Adjudication, and DLL-1 Concentration. **A** Flow chart of 317 patients enrolled at the Charité Benjamin Franklin emergency department; n = 300 serum samples were included for DLL-1 measurement. Expert adjudication classified patients into five groups: no infection, viral infection, bacterial infection, bacterial/viral co-infection, and sepsis-3 (based on Sepsis-3 criteria. **B** Box plots of serum DLL-1 concentration by group, shown as median (line), interquartile range (box), and minimum–maximum (whiskers)

Samples used in this analysis were obtained from patients presenting to the emergency department (ED) who participated in a prospective clinical study [10, 11] focused on the diagnosis of acute infections and sepsis, as depicted in Fig. 1. ED patients with a suspected acute infection were enrolled. For inclusion, patients were identified by trained ED personnel when an acute infection was among the primary differential diagnoses. Recruitment occurred at the time of initial ED presentation, and serum samples were collected with the first routine laboratory tests. Blood was drawn into 9 mL vacuum gel tubes, centrifuged at 3,000 × g for 10 min to isolate serum, and stored at –80 °C. After completion of the prospective studies, samples meeting predefined quality and volume criteria were selected, resulting in a final cohort of 300 patients.

### Trial endpoints

Each patient case was independently reviewed by a panel of two clinical experts to determine study outcomes, as previously described in detail [10, 11]. In brief, the adjudication process incorporated comprehensive clinical information, including radiologic findings, laboratory data (e.g., C-reactive protein (CRP) and procalcitonin levels), microbiological results, and follow-up records. Based on this evidence, the panel classified each patient as having a bacterial and/or viral infection or no infection at the time of presentation to the ED. Sepsis was adjudicated according to Sepsis-3 criteria. SOFA scores were derived from available laboratory, vital sign, and clinical data, and sepsis was defined as an acute increase of ≥ 2 SOFA points within 72 h after admission or a documented discharge diagnosis of sepsis [12]. This approach was chosen to capture both sepsis present at ED presentation and cases with early progression of organ dysfunction during the initial clinical course. All cases underwent expert review to confirm infection status and to avoid misclassification due to substantial baseline organ dysfunction (e.g., chronic kidney disease). Baseline organ function was systematically assessed for all patients based on clinical information, including pre-existing comorbidities and prior laboratory data when available. This allowed differentiation between acute organ dysfunction and chronic baseline impairment during endpoint adjudication. Follow-up assessments were conducted at 28 days after enrollment to determine survival status.

### *Measurement of DLL-1 via ELISA*

A commercially available enzyme-linked immunosorbent assay (ELISA) was used to measure DLL-1 levels (#ELH-DLL1, RayBiotech, Peachtree Corners, GA, USA). The samples were prepared according to the manufacturer’s instructions and diluted 30-fold for optimal analysis using the provided assay diluent. Measurements were conducted using an Infinite^®^ 200 Pro microplate reader (Tecan, Maennedorf, Switzerland), and concentration calculation was performed on the basis of the standard curve measurements.

### Statistical analysis

The statistical analysis for all the results shown in this study was performed in R version 4.3.0 [13] in RStudio Version 2024.04.0 + 735. Continuous variables are presented as medians with interquartile ranges and were compared via Mann‒Whitney U tests. Nominal variables are shown with frequencies and column percentages. The accuracy of the parameters is shown as the area under the receiver operating characteristic curve (AUROC), with 95% confidence intervals (CIs) calculated via DeLong’s method [14]. Comparisons between AUROCs were performed using DeLong’s test for correlated receiver operating characteristic curves. To assess the intercorrelations between the markers, scatterplots of each pair of markers were generated and approximated with a linear model, and a correlation matrix of Pearson’s coefficients was generated via the R package corrplot. Nonsignificant correlations, defined with a threshold of alpha = 0.01, were removed from the visualization. The study was conducted and reported in accordance with the STARD (Standards for Reporting of Diagnostic Accuracy Studies) guidelines.

For language editing and stylistic refinement, the authors used an AI-based language model (ChatGPT, version 5.2; OpenAI). The authors take full responsibility for the scientific content, data interpretation, and conclusions. In addition, the manuscript was edited for English language and grammar by Rubriq, an AI-powered tool (AJE – American Journal Experts, Winter Park, FL, USA).

## Results

A total of 300 patients presenting to the ED with clinically suspected infection were included for the analysis of DLL-1. The cohort has been described previously in detail [10, 11]. Based on the infection status adjudicated by an expert panel and Sepsis-3 criteria, 74 patients (24.7%) were classified as having sepsis. 194 patients (64.7%) were diagnosed with nonseptic infection, and infection was ruled out in 32 patients (10.7%). Among patients with nonseptic infection, 130 patients (43.3%) had bacterial infection, 39 patients (13.0%) had viral infection, and 25 patients (8.3%) had bacterial-viral coinfection (Fig. 1). During the 28-day follow-up, 18 patients (6.0%) died, including 14 patients with sepsis and 4 patients without sepsis. Compared with patients without sepsis, patients with sepsis were older and more frequently male and showed a higher prevalence of selected comorbidities, particularly chronic obstructive pulmonary disease, diabetes mellitus, and malignancy, whereas immunosuppression was numerically less frequent in the sepsis group (Table 1).

**Table 1:** Baseline Characteristics

	No Sepsis (n = 226)	Sepsis (n = 74)
Demographics		
Age [years]	71.5 (54.2–80.0)	75.0 (67.0–81.0)
Female Sex [%]	97 (42.9%)	29 (39.2%)
Malignant Disease [%]	59 (26.1%)	24 (32.4%)
Type 2 Diabetes [%]	41 (18.1%)	18 (24.3%)
COPD [%]	19 (8.4%)	16 (21.6%)
Immunosuppression [%]	52 (23.0%)	12 (16.2%)
Vital Signs		
Respiratory Rate [/min]	20 (17–24); n = 219	24 (20–30); n = 72
Systolic Pressure [mmHg]	125 (111–141); n = 225	110 (94–134)
Temperature [°C]	38 (37–39); n = 225	38 (37–39)
Heart Rate [/min]	98 (84–116)	105 (84–127); n = 73
Clinical Scores		
SOFA Score	1.0 (0.0–2.0)	4.5 (3.0–6.0)
NEWS2	3.0 (1.0–5.0)	7.0 (5.0–10.0)
Biomarkers		
DLL-1 [pg/mL]	8,085 (5591–11,059)	11,454 (8471–15,411)
C-reactive protein [mg/L]	63.6 (16.6–145.6)	126.7 (43.3–260.4)
Procalcitonin [μg/L]	0.2 (0.1–0.6); n = 222	1.5 (0.3–5.9); n = 73
WBC [10^9^ cells/L]	10.3 (7.8–14.3)	13.3 (8.3–18.8)
Lactate [mmol/L]	1.7 (1.3–2.1); n = 216	2.6 (1.8–3.7); n = 73
Study Outcomes		
28-day mortality	4 (1.8%)	14 (18.9%)

Baseline characteristics at enrollment. Nominal variables are shown with frequency and percentage; continuous variables are shown as median and interquartile range. The number of cases with valid data is shown for variables with missing data (e.g. n = 216 patients in the “No Sepsis” group had lactate concentration measurements).

### Discriminatory performance of DLL-1 and established biomarkers

**Fig. 2:**
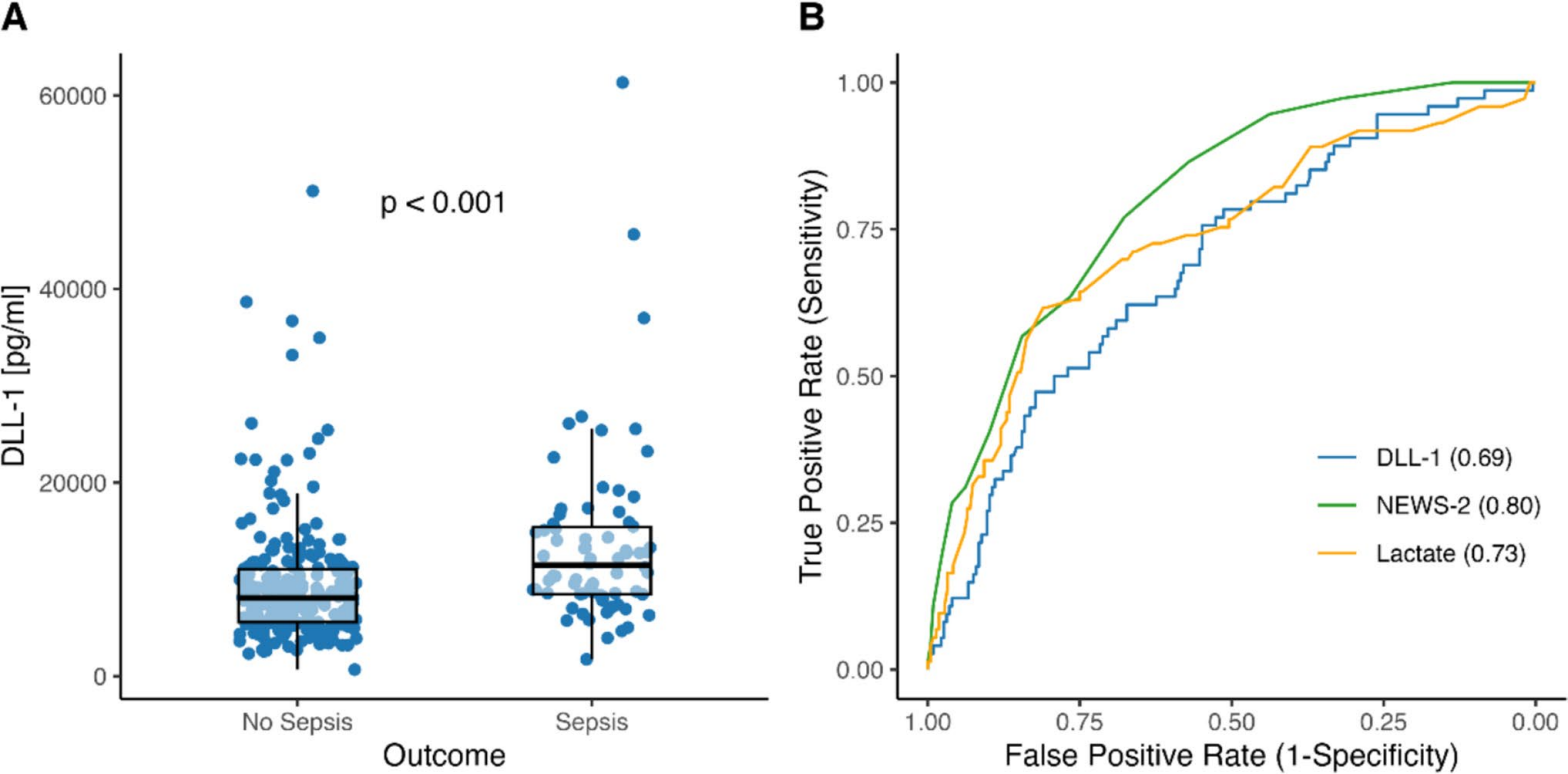
DLL-1 Concentrations and AUROC for Sepsis. **A** Box plot of serum DLL-1 levels in patients with and without sepsis, shown as median (line), interquartile range (box), and minimum–maximum (whiskers); p-value from Mann‒Whitney U test. **B** ROC curves for Sepsis with AUROC values for DLL-1 (blue), NEWS2 (green), and lactate (orange)

As shown in Fig. 2, the median concentration of DLL-1 for patients with sepsis was significantly higher (11,454 pg/mL) than that for the group without sepsis (8,085 pg/mL; p < 0.001). The median DLL-1 concentration among patients who died within 28 days was 9,631 pg/mL (Supplementary Table S1 and S2, Figure S1). The discriminatory performance of DLL-1 for identifying sepsis at ED presentation was moderate, with an AUROC of 0.69 (95% CI 0.62–0.76). The AUROC of DLL-1 did not differ significantly from that of CRP (AUROC 0.65, p = 0.371) or lactate (AUROC 0.73, p = 0.334) but was significantly lower than that of procalcitonin (AUROC 0.78, p = 0.041). NEWS2 showed the highest discriminatory performance (AUROC 0.80, 95% CI 0.75–0.85) and outperformed DLL-1 (p = 0.011). Of note, the diagnostic performance of DLL-1 for discriminating between bacterial and viral infection was low (AUROC 0.54), in contrast to procalcitonin and CRP (both AUROC 0.80) (Supplementary Table S3a and Figure S2). Within the subgroup of patients with bacterial infection, the diagnostic performance of DLL-1 for identifying sepsis was moderate (AUROC 0.66) and did not differ significantly from that of procalcitonin (AUROC 0.75) or CRP (AUROC 0.57) (Supplementary Table S3b).

### Exploratory cut-off analysis

In an exploratory analysis, predefined and data-driven cut-off values for DLL-1 and established biomarkers were evaluated for the identification of sepsis (Table 2). For DLL-1, a Youden index–derived threshold of 8,452 pg/mL yielded a sensitivity of 75.7% and a specificity of 54.9%. When applying a higher DLL-1 cut-off of 10,623 pg/mL, as previously suggested by Hölle et al. [15], the sensitivity decreased to 54.1%, while the specificity increased to 73.5%. Procalcitonin, at the established clinical cut-off of 0.5 µg/L, showed a sensitivity of 67.2% and a specificity of 70.3%. CRP demonstrated high sensitivity at lower cut-off values with limited specificity, whereas higher thresholds were associated with reduced sensitivity. Lactate evaluated at the routinely used cut-off of 20 mg/dL (2.2 mmol/L) showed a specificity of 75.9% with a sensitivity of 63.0%.

**Table 2:** Exploratory cut-off analysis

**Biomarker**	Threshold	Sensitivity	Specificity
DLL-1 [pg/mL]	8,452	75.68	54.87
DLL-1 [pg/mL]	10,623*	54.05	73.45
Procalcitonin [mg/L]	0.5	67.12	70.27
CRP [mg/dL]	20	87.84	27.88
CRP [mg/dL]	50	71.62	44.25
CRP [mg/dL]	100	55.41	62.39
Lactate [mmol/L]	2.2	63.01	75.93

*The DLL-1 cut-off of 10,623 pg/mL was previously suggested by Hölle et al.

Sensitivity and specificity of DLL-1 and established biomarkers for sepsis identification at ED presentation using predefined clinical cut-offs and a Youden index–derived threshold for DLL-1.

### DLL-1 concentrations according to infection site

**Fig. 3:**
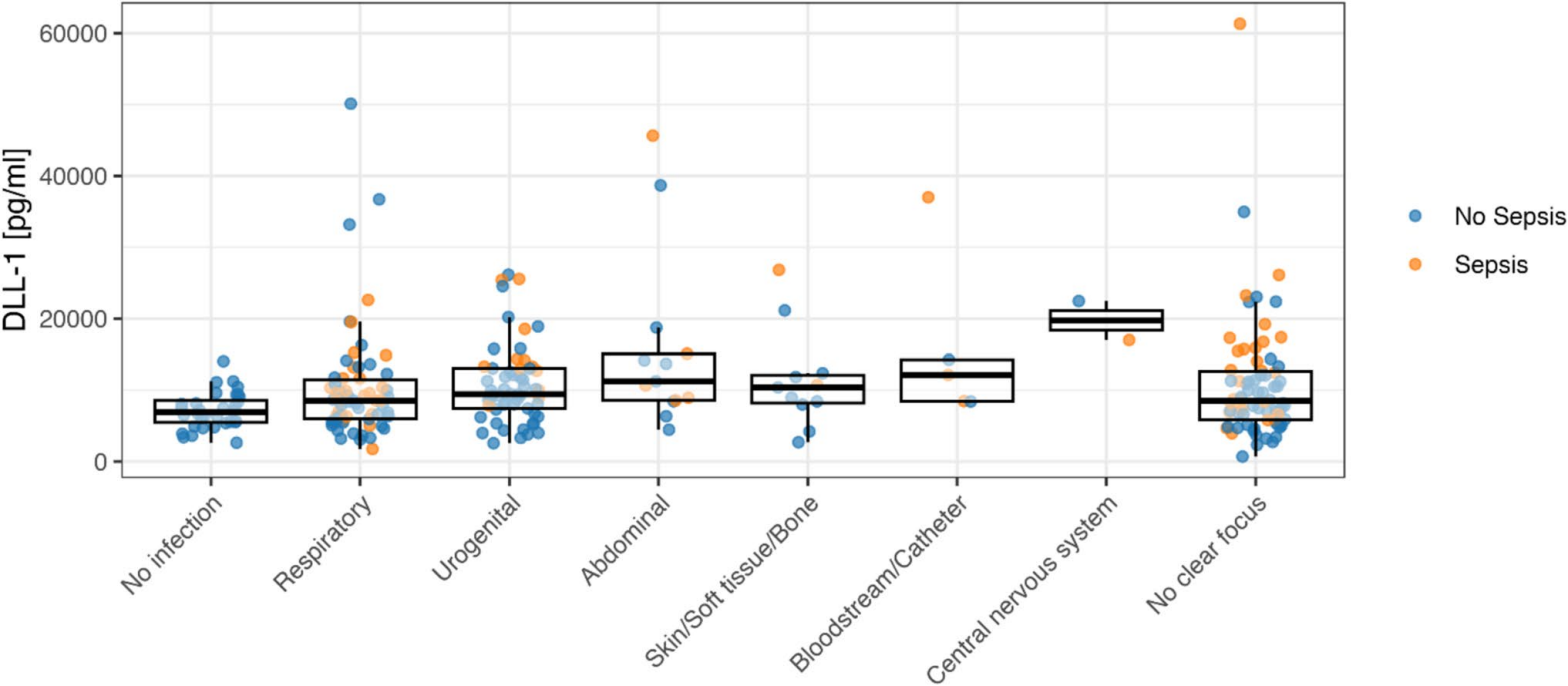
DLL-1 Levels by Infection Focus. Box plots of serum DLL-1 concentration in non-septic (blue) and septic (orange) cases by infection site, shown as median (line), interquartile range (box), and minimum–maximum (whiskers). “No clear focus” indicates cases where multiple infection sites were present or in which no definite infection focus could be identified

DLL-1 concentrations stratified by infection focus and sepsis status are shown in Fig. 3. DLL-1 values displayed substantial within-focus variability and considerable overlap between patients with sepsis and without sepsis across infection sites. While higher DLL-1 concentrations were more frequently observed among septic patients in several foci (e.g., abdominal infection and primary bacteremia), marked overlap and outliers were present, particularly in pulmonary infections. Of note, an individual nonseptic patient exhibited the highest observed DLL-1 concentration (Fig. 3). Detailed DLL-1 concentrations by infection focus are provided in Supplementary Table S4. In addition, median DLL-1 concentrations were significantly higher in patients with positive blood cultures compared with those with negative blood cultures (11,497 pg/mL vs. 8,016 pg/mL; p < 0.001 Supplementary Figure S3).

### Correlation of DLL-1 with clinical and laboratory parameters

**Fig. 4:**
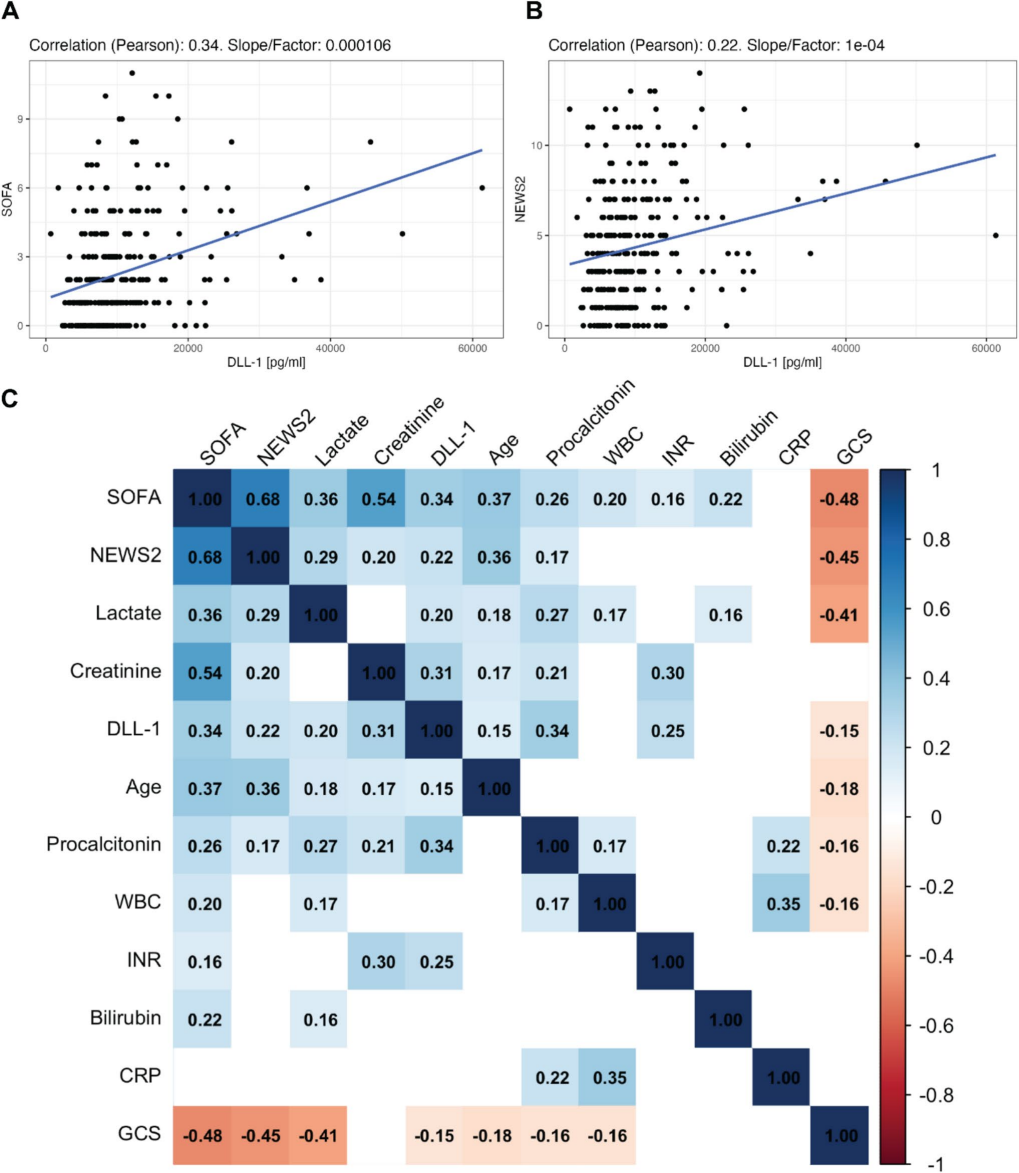
Correlation of DLL-1 with established parameters. **A**, **B** Scatterplots of DLL-1 vs. SOFA (**A**) and NEWS2 (**B**) and linear regression (blue); Pearson’s correlation shown. **C** Pearson’s correlation matrix of DLL-1, standard biomarkers and clinical scores. Nonsignificant correlations, defined as p > 0.01, are blank

DLL-1 concentrations showed weak to moderate correlations with selected clinical and laboratory parameters (Fig. 4). Positive correlations were observed between DLL-1 and markers of disease severity and organ dysfunction, including lactate levels and SOFA-related parameters, whereas correlations with conventional inflammatory markers such as C-reactive protein and white blood cell count were weaker. Procalcitonin showed a moderate correlation with DLL-1, comparable in magnitude to the correlation observed with the SOFA score. DLL-1 also showed a moderate correlation with serum creatinine levels (r = 0.31). Consistent with this finding, DLL-1 concentrations were higher in patients with chronic kidney disease (CKD) compared with those without CKD; however, within both groups, DLL-1 levels remained higher in patients with sepsis. Detailed results are shown in Supplementary Fig. S4. DLL-1 demonstrated limited correlation with vital signs and demographic variables, suggesting that DLL-1 reflects distinct aspects of the host response not captured by routine clinical or laboratory measures.

## Discussion

In this study, we aimed to determine whether DLL-1 can aid in identifying patients in the emergency department (ED) with sepsis or at risk of deterioration into sepsis. To address this question, we examined DLL-1 concentrations in samples derived from a prospective ED cohort. Patients with clinically suspected acute infection were enrolled at presentation, and blood was obtained during the initial clinical assessment. In this setting, DLL-1 concentrations were significantly higher in patients who had sepsis or developed sepsis within 72 h, although the overall diagnostic performance was moderate. This definition reflects a combined diagnostic and early risk stratification approach, acknowledging that in the ED, sepsis may already be present at presentation or evolve shortly thereafter.

### Comparison with previous ICU-based studies

Previous investigations of DLL-1 have largely focused on hospitalized and critically ill patients with established sepsis or septic shock, predominantly in intensive care unit settings. In these cohorts, DLL-1 concentrations were higher and demonstrated stronger associations with disease severity and mortality, reflecting advanced stages of systemic inflammation and organ dysfunction. Notably, neither the study by Theobald et al. nor the analysis by Hölle et al. evaluated DLL-1 at the time of ED presentation in patients with undifferentiated clinical suspicion of infection [9, 15].

In contrast, our study assessed DLL-1 at a substantially earlier clinical decision point, at which patients present across a broad spectrum of severity ranging from early stages of dysregulated host response to clinically manifest sepsis. This difference in disease stage is reflected by lower overall organ dysfunction at baseline in our cohort, with a median SOFA score of 4.5 (IQR 3–6) among patients classified as septic, compared with a median SOFA score of 7 (IQR 4–10) reported by Hölle et al. and a mean SOFA score of 7.3 (± 2.8) in the ICU cohort described by Theobald et al. [9, 15].

Unlike ICU cohorts, where patients are typically admitted with a confirmed or highly suspected diagnosis of sepsis, ED populations comprise individuals in whom both the presence and trajectory of sepsis still need to be determined. Notably, the observed moderate diagnostic performance of DLL-1 is consistent with the intrinsic diagnostic uncertainty of early emergency care. These findings suggest that the diagnostic characteristics of DLL-1 are influenced by disease stage and the timing of biomarker assessment and that performance metrics derived from intensive care unit populations cannot be directly extrapolated to early emergency department risk stratification.

### Orthogonality and implications for composite biomarker strategies

An additional relevant observation is the correlation pattern of DLL-1 with routinely available clinical and laboratory parameters. DLL-1 showed a moderate correlation with both SOFA score and procalcitonin (r = 0.34 for each), whereas correlations with CRP, white blood cell count, and bilirubin were nonsignificant (p > 0.01). Importantly, the association with SOFA was more pronounced than with the NEWS2, which primarily reflects acute physiological instability rather than established organ dysfunction. This distinction is biologically plausible, as DLL-1-mediated Notch signaling has been implicated in endothelial activation and microvascular dysfunction processes that are more directly captured by organ dysfunction scores such as SOFA than by vital-sign-based early warning scores. The observed correlation pattern therefore suggests that DLL-1 is more closely linked to infection-associated organ involvement than to transient physiological derangements alone.

From a clinical and translational perspective, these findings support the concept that DLL-1 is unlikely to function as a stand-alone “single-marker solution” for sepsis but may add value as part of composite risk stratification approaches. Given the biological heterogeneity of sepsis, a more robust strategy may involve integrating biomarkers that reflect complementary pathophysiological domains—such as systemic inflammation, metabolic derangement, and endothelial dysfunction—together with clinical scores [16]. In this framework, DLL-1 may represent a candidate marker contributing an endothelial- and Notch-related dimension of the host response [4].

DLL-1 showed limited ability to discriminate between bacterial and viral infections. This supports the concept that DLL-1 reflects host-response and organ dysfunction per se rather than pathogen-specific signals. In line with this, higher DLL-1 concentrations in patients with positive blood cultures should not be interpreted as pathogen specificity, but instead as a reflection of a more intense systemic host-response. Blood culture positivity likely represents a surrogate of invasive infection and increased host–pathogen interaction, which may be associated with enhanced host-response pathways captured by DLL-1. Within bacterial infections, DLL-1 demonstrated moderate performance for identifying sepsis, further supporting its role as a host-response marker.

### DLL-1 in the context of renal dysfunction

DLL-1 showed a moderate correlation with serum creatinine levels. In addition, patients with pre-existing chronic kidney disease (CKD) exhibited higher DLL-1 concentrations compared with those without CKD (Supplementary Fig. S4). However, DLL-1 levels were also higher in patients with sepsis irrespective of underlying renal function (Supplementary Fig. S4). These findings suggest that renal dysfunction may contribute to elevated baseline DLL-1 levels, potentially reflecting altered clearance or accumulation [2, 17]. At the same time, the further increase in DLL-1 concentrations observed in septic patients indicates that DLL-1 is not solely driven by renal function but likely reflects additional pathophysiological processes related to the host response in sepsis.

Experimental studies suggest that DLL-1–mediated Notch signaling is involved in the regulation of monocyte differentiation and endothelial function [18]. In particular, DLL-1 has been implicated in the transition of Ly6C^high^ to Ly6C^low^ monocytes [19]. Chousterman et al. demonstrated a protective role of Ly6C^high^ monocytes in mitigating kidney damage during sepsis in mice [20]. These findings suggest a potential biological link between DLL-1 and immune and organ-specific responses during sepsis. Importantly, pre-existing renal dysfunction was carefully considered during endpoint adjudication to ensure accurate sepsis classification. Nevertheless, the present cohort does not allow reliable mechanistic attribution. It remains unclear whether elevated DLL-1 concentrations in patients with impaired renal function primarily reflect reduced renal clearance, accumulation, sepsis-associated kidney injury, or broader endothelial dysfunction. Further studies are required to better define the interaction between DLL-1, renal function, and sepsis and to clarify its clinical utility.

### Clinical implications

From a practical emergency care perspective, our findings underscore that DLL-1 should not be interpreted as a diagnostic substitute for established clinical assessment or sepsis screening tools. Nevertheless, its value may lie in supporting early clinical decision-making in situations characterized by diagnostic uncertainty, particularly among patients with suspected infection who do not yet fulfill overt sepsis criteria.

In the ED, early management decisions often need to be made before definitive organ dysfunction is apparent. In this context, a biomarker reflecting infection-associated host response and early organ involvement may help identify patients who warrant closer monitoring, expedited diagnostics, or early escalation of care. DLL-1 may therefore be most relevant in intermediate-risk scenarios, where routine clinical parameters and scores are inconclusive. In the exploratory cut-off analysis, DLL-1 showed a sensitivity of approximately 76% at a specificity of 55%. This corresponded to a positive likelihood ratio of approximately 1.7 and a negative likelihood ratio of 0.44. While this profile may suggest potential utility as a rule-out component, the overall diagnostic performance remains insufficient for use as a stand-alone test. Accordingly, DLL-1 may be best positioned as part of a multimodal approach rather than as an isolated test. Integration of DLL-1 with established biomarkers and clinical scoring systems could improve the identification of patients at risk for sepsis-related deterioration, particularly in scenarios where conventional parameters remain equivocal. Future studies should therefore focus on validating DLL-1 within combined biomarker panels and on assessing its incremental value for clinical decision-making in the ED.

### Strengths and limitations

Our study has strengths and limitations. DLL-1 was evaluated in a well-characterized, prospectively enrolled ED cohort that reflects the full clinical spectrum of patients presenting with suspected infection, including early disease stages prior to intensive care unit admission. Biomarker measurements were obtained at the time of initial clinical assessment, corresponding to the earliest possible decision point in emergency care. Outcome adjudication was performed by an expert panel using a stringent Sepsis-3–based definition, with systematic assessment of baseline organ function for all patients and careful consideration of pre-existing organ dysfunction, thereby reducing the risk of misclassification.

Several limitations should be acknowledged. First, this was a single-center study, which may limit generalizability. Second, outcome adjudication was not blinded to routinely available biomarkers such as CRP, procalcitonin, and microbiological results, which were part of the clinical information explicitly used to define infection and sepsis. This introduces the potential for incorporation bias, which may favor established biomarkers in comparative analyses, as these parameters contributed to the reference standard. Third, DLL-1 was assessed at a single time point, and dynamic changes over time were not captured. Fourth, the study includes both patients with sepsis at the time of ED presentation and a small number of patients who developed sepsis within 72 h. While the majority of cases fulfilled sepsis criteria at presentation, the exact timing of organ dysfunction onset was not systematically recorded, precluding a formal subgroup analysis. Therefore, the analysis reflects a combined diagnostic and early risk stratification approach, which corresponds to the clinical reality in the ED, where sepsis may either already be present or evolve shortly after initial assessment.

Given the relatively small number of patients with sepsis included in this study, the findings should be interpreted with caution and considered hypothesis-generating. Larger multicenter studies are required to validate the diagnostic performance of DLL-1 to assess its role in clinical decision-making across diverse ED populations. Further, only 74 patients fulfilled the sepsis endpoint, the cut-off analysis should also be interpreted cautiously.

DLL-1 measurements in the present study were performed using a laboratory-based ELISA requiring serum processing and analysis. This approach is currently not compatible with rapid bedside decision-making in the ED, where timely results are essential. Together with other studies our results may facilitate approaches for the development of a rapid point-of-care or whole-blood assay.

## Conclusions

In this emergency department cohort of patients with suspected infection, DLL-1 concentrations were significantly higher in patients with sepsis, but demonstrated only moderate diagnostic performance when measured at initial presentation. This finding is consistent with earlier disease stages, lower degrees of organ dysfunction, and greater diagnostic uncertainty compared with ICU populations. DLL-1 may provide complementary host-response information beyond established biomarkers, but does not support stand-alone use for early sepsis diagnosis and requires further evaluation within multimodal risk stratification strategies.

## Supplementary Information

Below is the link to the electronic supplementary material.

Supplementary file1 (DOCX 509 kb)

## Abbreviations

AUROC: Area under the receiver operating characteristic
CI: Confidence interval
COPD: Chronic obstructive pulmonary disease
CKD: Chronic kidney disease
CRP: C-reactive protein
DLL-1: Delta-like canonical Notch ligand-1
ED: Emergency department
ELISA: Enzyme-linked immunosorbent assay
GCS: Glasgow Coma Scale
INR: International Normalized Ratio
IQR: Interquartile range
NEWS2: National early warning score version 2
ROC: Receiver operating characteristic
SOFA: Sequential failure assessment

## Declarations

### Data availability

The raw data can be obtained upon reasonable request from the corresponding author.
