# Supplementary material for: From bench to triage: diagnostic utility of delta-like canonical notch ligand-1 (DLL-1) for early sepsis prediction in the emergency department
Source: Infection. 2026 Apr 13;54(3):1547–57. doi: 10.1007/s15010-026-02794-y (PMC13323347; doi:10.1007/s15010-026-02794-y)
Supplement: Supplementary file 1 — Supplementary file1 (DOCX 509 kb) [file 15010_2026_2794_MOESM1_ESM.docx]

# Supplement: From Bench to Triage: Diagnostic Utility of Delta-like Canonical Notch Ligand-1 (DLL-1) for Early Sepsis Prediction in the Emergency Department

Aurelia Hübner^1^, Katharina Friedrich^2^, Noa Galtung^1^, Vivienne Theobald^3^, Judith Schenz^3^, Britta Hecke^2^, Melanie Kraß^2^, Markus A. Weigand^3^, Kai Kappert^2^, Wolfgang Bauer^1*^

# Supplementary Table S1. Biomarker levels for 28-day mortality

| **Biomarker** | Survived (Median [IQR]) | 28-day mortality (Median [IQR]) |
| --- | --- | --- |
| DLL-1 [pg/mL] | 8,603 (6,241-12,013) | 9,631 (7,598-16,367) |
| Procalcitonin [mg/L] | 0.27 (0.11-0.89) | 1.71 (0.42-12.16) |
| CRP [mg/dL] | 68.1 (20.5-157.8) | 189.1 (61.8-260.5) |
| WBC [10^9^cells/L] | 11.0 (7.9-14.8) | 14.5 (9.1-19.8) |
| Lactate [mmol/L] | 1.78 (1.39-2.33) | 2.90 (2.22-4.09) |
| NEWS2 | 4 (1-6) | 7.5 (5-10) |

Median concentrations (interquartile range) of DLL-1, established biomarkers, and NEWS2 stratified by 28-day survival status. WBC, white blood cell count. CRP, C-reactive protein.

**Supplementary Table S2. Prognostic performance for 28-day mortality**

| **Biomarker** | AUROC | 95% CI | p value vs. DLL-1 |
| --- | --- | --- | --- |
| DLL-1 | 0.60 | 0.45-0.75 | - |
| Procalcitonin | 0.72 | 0.58-0.86 | 0.127 |
| CRP | 0.68 | 0.55-0.82 | 0.369 |
| WBC | 0.63 | 0.48-0.79 | 0.745 |
| Lactate | 0.76 | 0.64-0.88 | 0.014 |
| NEWS2 | 0.76 | 0.65-0.86 | 0.047 |

Area under the receiver operating characteristic curve (AUROC) for DLL-1 and established biomarkers for prediction of 28-day mortality. P values indicate comparison of AUROCs with DLL-1 using DeLong’s test.

**Supplementary Table S3a. Prognostic performance for bacterial vs. viral infection**

| **Biomarker** | AUROC | 95% CI | p value vs. DLL-1 |
| --- | --- | --- | --- |
| DLL-1 | 0.54 | 0.45-0.63 | - |
| Procalcitonin | 0.80 | 0.74-0.87 | <0.001 |
| CRP | 0.80 | 0.74-0.87 | <0.001 |

Area under the receiver operating characteristic curve (AUROC) for DLL-1 and established biomarkers for prediction of bacterial infection. P values indicate comparison of AUROCs with DLL-1 using DeLong’s test.

**Supplementary Table S3b. Prognostic performance for sepsis vs. no sepsis in bacterial infections**

| **Biomarker** | AUROC | 95% CI | p value vs. DLL-1 |
| --- | --- | --- | --- |
| DLL-1 | 0.66 | 0.57-0.74 | - |
| Procalcitonin | 0.75 | 0.66-0.83 | 0.084 |
| CRP | 0.57 | 0.47-0.66 | 0.163 |

Area under the receiver operating characteristic curve (AUROC) for DLL-1 and established biomarkers. P values indicate comparison of AUROCs with D LL-1 using DeLong’s test.

**Supplementary Table S4. DLL-1 concentrations by infection focus**

| **Focus of infection** | n | Median [pg/mL] (IQR) |
| --- | --- | --- |
| No infection | 32 | 6,916 (5,477- 8,539) |
| Respiratory | 66 | 8,518 (6,003-11,444) |
| Urogenital | 57 | 9,435 (7,431-13,031) |
| Abdominal | 13 | 11,221 (8,567-15,078) |
| Skin/Soft tissue/Bone | 11 | 10,384 (8,204-12,092) |
| Bloodstream/Catheter | 5 | 12,131 (8,460-14,243) |
| Central nervous system | 2 | 19,711 (18,345-21,076) |
| No clear focus | 74 | 8,488 (5,846-12,627) |

Values are presented as median and interquartile range (IQR) for all patients included. “No clear focus” indicates cases in which no definite infection source could be identified or where multiple infection sites were present.

**Supplementary Fig. S1**

#
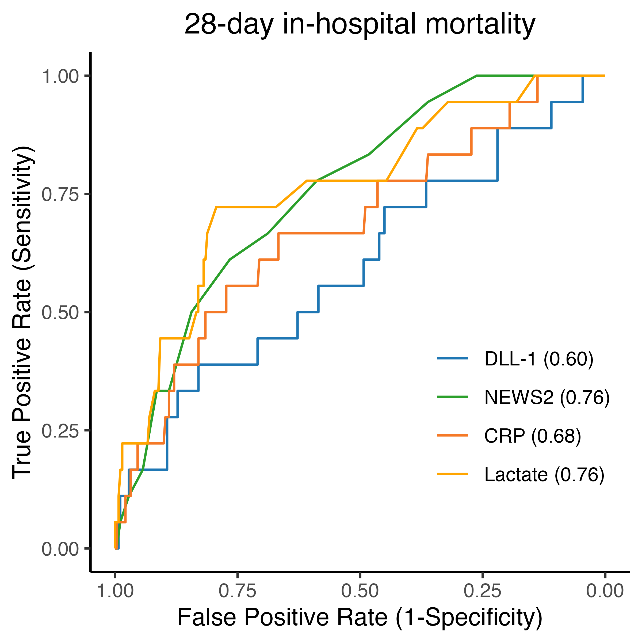


AUROC for endpoint 28-day mortality. ROC curves for 28-day mortality with AUROC values for DLL-1 (blue), NEWS-2 (green), CRP (orange), and lactate (yellow).

**Supplementary Fig. S2**


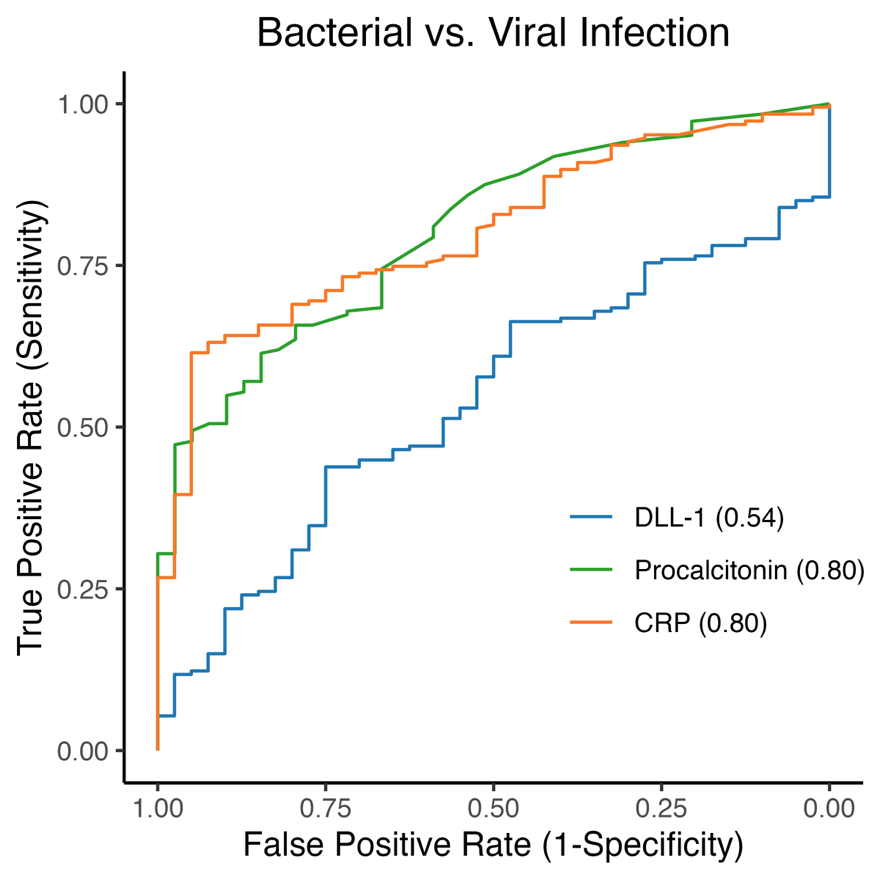


AUROC for endpoint bacterial infection vs. viral infection. ROC curves for bacterial infection with AUROC values for DLL-1 (blue), Procalcitonin (green), and CRP (orange).

**Supplementary Fig. S3**


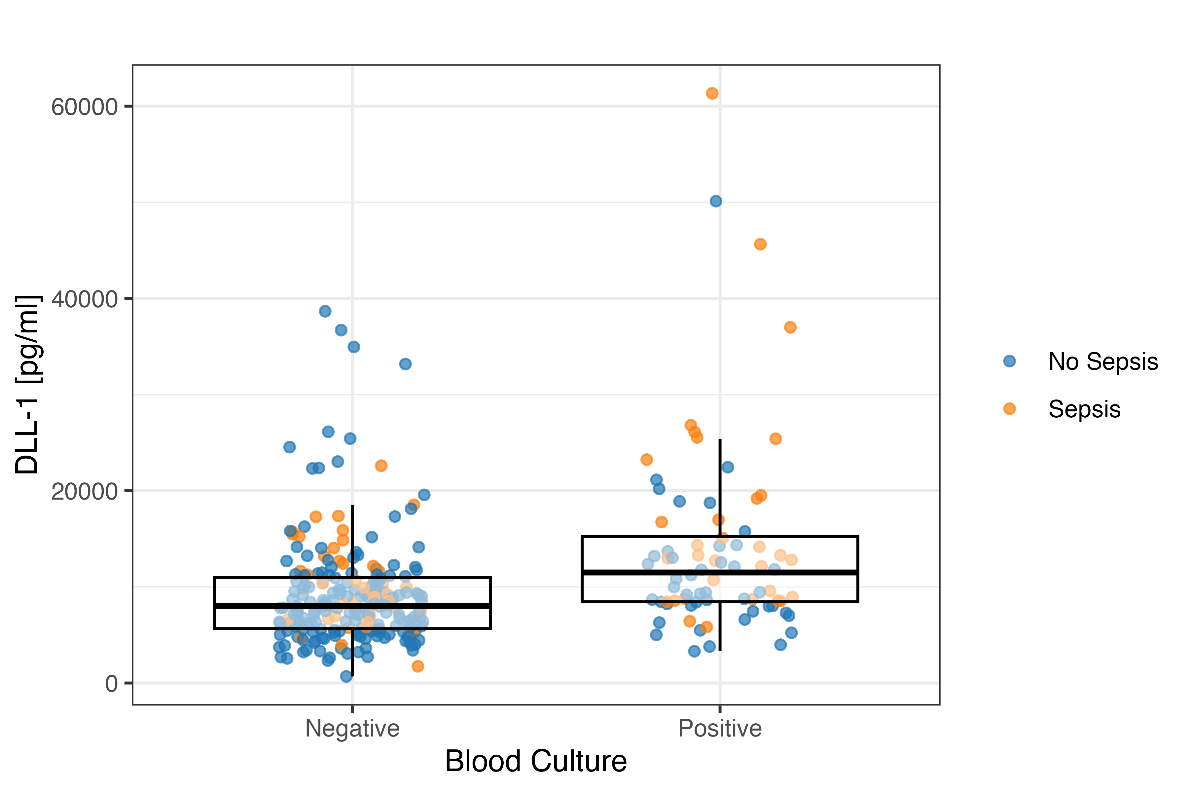


DLL-1 concentrations by blood culture status**.** DLL-1 concentrations in patients with positive versus negative blood cultures at emergency department presentation, shown as individual data points (blue: non-septic, orange: septic patients) with median and interquartile range.

**Supplementary Fig. S4**
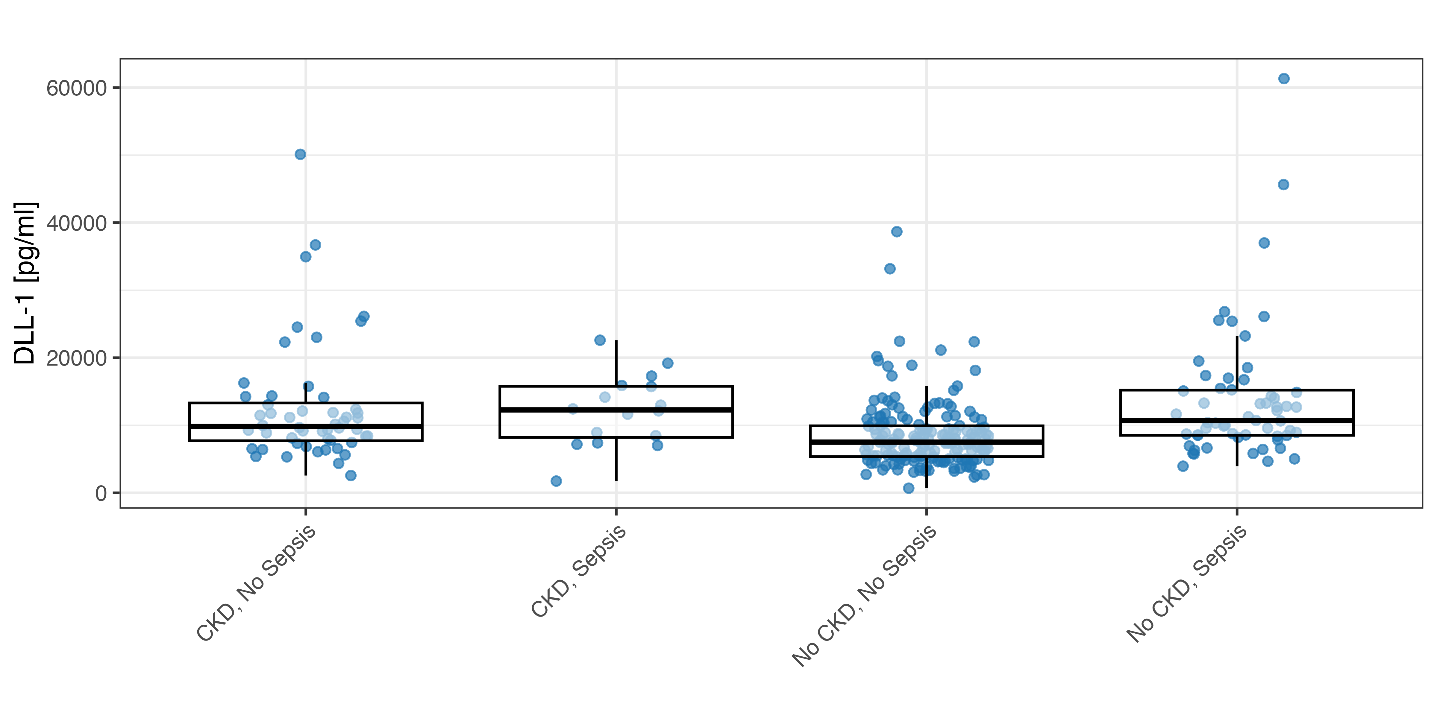
DLL-1 concentrations according to chronic kidney disease status. DLL-1 concentrations stratified by the presence of chronic kidney disease (CKD) and sepsis, shown as individual data points with median and interquartile range. Patients were categorized into four groups: CKD without sepsis, CKD with sepsis, no CKD without sepsis, and no CKD with sepsis. Patients with CKD and without sepsis (n=52) showed DLL-1 plasma levels of 9,821 pg/mL (IQR 7,711–13,306). Patients with CKD and sepsis (n=16) had higher DLL-1 levels of 12,279 pg/mL (IQR 8,193–15,773). Patients without CKD and without sepsis (n=174) showed DLL-1 concentrations of 7,504 pg/mL (IQR 5,351–9,923), whereas patients without CKD and with sepsis (n=58) had levels of 10,684 pg/mL (IQR 8,513–15,204). These analyses are descriptive and exploratory and were not intended for formal statistical comparison.
